# DEMENTIA SUBTYPES AND LIVING WELL IN PEOPLE WITH MILD TO MODERATE DEMENTIA. LONGITUDINAL FINDINGS OVER SIX YEARS

**DOI:** 10.1093/geroni/igad104.1430

**Published:** 2023-12-21

**Authors:** Linda Clare, Anthony Martyr, Laura Gamble, Fiona Matthews

**Affiliations:** University of Exeter Medical School, Exeter, England, United Kingdom; University of Exeter, Exeter, England, United Kingdom; Newcastle University, Newcastle upon Tyne, England, United Kingdom; Newcastle University, Newcastle upon Tyne, England, United Kingdom

## Abstract

The heterogeneity of symptoms across dementia subtypes has important implications for clinical practice and dementia research. Variations in subtypes and associated symptoms may influence capability to ‘live well’ for people with dementia. In this paper we investigate the potential impact of dementia subtypes on the quality of life of people with dementia. We used longitudinal data from 1749 individuals with mild-to-moderate dementia participating in the IDEAL and IDEAL-2 studies. Assessments took place on entry and were followed up at 12, 24, 48, 72 and 96 months. We examined change in quality of life post diagnosis and associations with dementia subtypes using mixed effects models. Slightly more than half of participants , 52.4%, had a diagnosis of Alzheimer’s disease, 10.2% vascular dementia and 19.8% mixed Alzheimer’s and vascular dementia. Rarer subtypes of dementia, frontotemporal, Parkinson’s type, Lewy bodies accounted for 17.6% of participants . Dementia subtype had the greatest associations with quality of life at the time of diagnosis. Compared to individuals with Alzheimer’s disease, those with Parkinson’s disease dementia (-4.89, 95% CI: -6.60 to -3.417) and dementia with Lewy bodies (-4.90, 95% CI: -6.38 to -3.42) had relatively poorer quality of life at baseline. The difference in quality of life for people with vascular dementia (-1.70, 95% CI: -3.55 to -1. 35) and mixed Alzheimer’s and vascular dementia (-1.70 95% CI:-2.47 to -0.92) was less marked. There was little change in quality of life longitudinally and no difference in quality-of-life trajectories by dementia subtype

